# Merkel cell polyomavirus in Merkel cell carcinoma of Italian patients

**DOI:** 10.1186/1743-422X-8-103

**Published:** 2011-03-07

**Authors:** Francesca Paolini, Pietro Donati, Ada Amantea, Stefania Bucher, Emilia Migliano, Aldo Venuti

**Affiliations:** 1 Laboratory of Virology, Regina Elena Cancer Institute Via delle Messi d'Oro 156 00158 Rome, Italy; 2 Laboratory of Cutaneous Histopathology, San Gallicano Dermatologic Institute I.R.C.C.S, Via Chianesi, 53, 00144 Rome, Italy; 3 Plastic Surgery Department, San Gallicano Dermatologic Institute I.R.C.C.S, Via Chianesi, 53, 00144 Rome, Italy

## Abstract

**Background:**

Merkel cell carcinoma (MCC) is a rare but very aggressive human malignancy of elderly or immunosuppressed patients. Clonal integration of a new human polyomavirus, the Merkel cell polyomavirus (MCPyV), has been reported in MCC patients. The main objective of the study was the detection of MCPyV and viral expression in clinical samples of Italian patients who were diagnosed MCC.

**Findings:**

DNA and RNA were extracted from nine MCCs to detect the presence of MCPyV. Viral large T gene (LT1 and LT3), and viral capsid gene (VP1) were detected by polymerase chain reaction (PCR) based methods, and the amplified PCR products were subjected to direct sequencing. The presence of viral T antigen and/or viral capsid DNA sequences was demonstrated in eight of the nine MCC lesions, whereas RNA transcripts were detected in three MCCs.

**Conclusions:**

These findings indicate a potential role of MCPyV in the pathogenesis of at least a subset of MCCs.

## Findings

Merkel cell carcinoma (MCC) is a rare but aggressive human skin cancer that often appears in the older white population. Sun exposure and immunosuppression are likely to play a significant pathogenetic role [1,2]. Management of MCC is controversial, most of patients are treated by surgical excision with sentinel lymph node biopsy, followed by irradiation [3]. Conventional adjuvant chemotherapy lacks evidence of survival benefit and may be associated with poorer outcomes [4]. Using digital transcriptome subtraction Feng et al. [5] reported PCR detection of Merkel cell polyomavirus (MCPyV) in most MCC specimens, and clonal integration of the viral genome was identified, suggesting a role for the virus in the pathogenesis of this skin cancer. The MCPyV is a small polyomavirus with a circular DNA encoding a T antigen oncoprotein locus [5]. The detection frequency of MCPyV DNA in MCC seems not to correlate with age, sex, histological subtype of carcinoma, or the time period during which the cancer was detected. In addition it is unclear whether integration of MCPyV DNA into the host genome is associated with some subtype of MCC. Nevertheless this infection could be considered a determinant clinical factor of this rare tumour [6].

**Figure 1:**
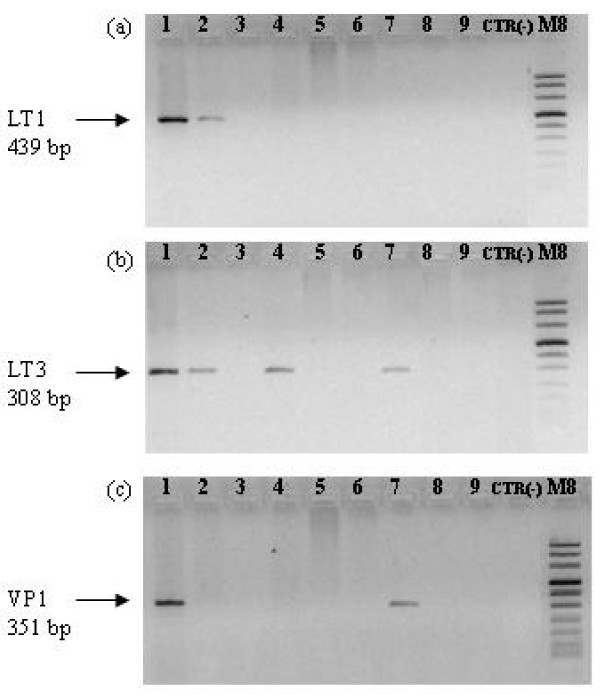
**Amplification of LT1 (a), LT3 (b) and VP1 (c) viral genes**. PCR was performed with MCPyV specific primers; the numbers indicate the patients as in Table 1. M8, molecular weight marker (Roche, Milan, Italy); CTR (-), sterile water as negative control.

The presence of MCPyV in MCC was already reported by several research groups [7-9] but data from Italian patients are lacking. The goal of this study was the detection of the presence and expression of MCPyV in a group of Italian MCC patients referring to our Institution for treatment. The study included formalin-fixed and paraffin-embedded (FFPE) resection specimens of 9 MCC from patients treated in our Institution. The mean age was 73 years, 6 males and 3 females. All tissue samples were collected for diagnostic purposes and an informed consent at the procedures, approved by the local Ethical Committee (Prot.n. CE/312/05), was obtained from all patients. Sections of 10 μm were obtained from FFPE tissue specimens of the patients. The sections were extracted with xylene to remove the paraffin, followed by two washes with absolute ethanol to remove the xylene. DNA and RNA were extracted by QIAamp DNA Mini kit and RNeasy Plus Mini kit (QIAGEN, Milan, Italy), respectively, according to the manufacturer's instructions. The presence of amplifiable DNA and RNA was confirmed by the amplification of human β-globin gene [10] and of human β-Actin gene, respectively. Primers for β-Actin were Act-up (5'-ACCACACCTTCTACAATGAGCTGCGTG-3') and Act-down (5'-CACAGCTTCTCCTTAATGTCACGCACG-3'). DNA, RNA and PCR mixtures were prepared and kept in separate rooms. For MCPyV DNA detection, the LT1, LT3, VP1 and M1/2, LT5, VP1.3, P1, P3, P6, P9, P12 LT2 primer sets were utilised [5]. In addition, the LT1 and M1/2 primer sets were used for nested PCR. All PCR mixtures consisted of 500 nM of each primer, 200 μM of each dNTP (Roche, Milan, Italy), 1 unit of thermostable Platinum Taq Polymerase, 1x reaction buffer (both from Invitrogen, Milan, Italy) and 1.5 mM MgCl_2_. Total RNA was pre-treated with DNase I (Deoxyribonuclease I, Amplification grade, Invitrogen, Milan, Italy) and tested by RT-PCR utilizing the "One step commercial kit" (Invitrogen, Milan, Italy) according to the manufacturer's instructions utilising LT1 and M1/2 primer sets for nested PCR. All amplification reactions were performed in a i-Cycler (Bio-Rad Laboratories, Milan, Italy). Aliquots of 15 μl from the PCR and RT-PCR products were submitted to electrophoresis in 2% ethidium bromide stained gel and were visualised under UV light. Sterile water without DNA or RNA template was used as PCR-negative controls. All the purified PCR products were subjected to direct sequencing in an automated apparatus (Biogen, Rome, Italy). DNA sequences were compared with the reference sequences of the National Center for Biotechnology Information (NCBI) Entrez Nucleotide database, using the NCBI Blast program. In order to ascertain the presence of episomal MCPyV MCC samples were analysed by the multiply primed rolling-circle amplification (RCA) method. This method utilizes the Ф29 DNA polymerase with random hexamer primers to amplify the circular DNA virus genomes without the need for prior knowledge of their DNA sequences. Multiply primed RCA was performed with the TempliPhi 100 amplification kit (Amersham, Biosciences, Milan, Italy) according to the manufacturer's instructions with 450 μM extra dNTPs. Negative control samples were made with buffer without the TempliPhi enzyme. Results from the above mentioned molecular analyses showed that eight tumours out of the nine MCC were positive for MCPyV DNA by PCR. Data obtained with the different primer sets are summarized in Table 1 and Figure 1. One MCC was tested positive to 9 MCPyV primer sets, two MCCs were positive to 4 MCPyV primer sets, one MCC was positive to 3 MCPyV primer sets, four MCCs were positive to 1 primer set and only one MCC was negative to all MCPyV primer sets.

**Table 1:** DNA, RNA, and RCA analysis for MCPyV detection

ID	Gender	DNA														RNA		RCA
		**β- globin**	**LT1**	**VP1**	**LT3**	**M1/2**	**LT5**	**VP 1.3**	**P1**	**P3**	**P6**	**P9**	**P 12**	**LT2**	**LT1 Nested M1/2**	**β- actin**	**LT1 Nested M1/2**	

1	M	+	+	+	+	+	-	+	+	-	+	+	-	-	+	+	+	-

2	F	+	+	-	+	-	-	-	+	-	-	-	-	-	+	+	+	-

3	M	+	-	-	-	-	-	-	-	-	-	-	-	-	+	-	-	+

4	M	+	-	-	+	-	-	-	+	-	-	+	-	-	-	+	-	-

5	M	+	-	-	-	-	-	-	-	-	-	-	-	-	+	+	-	-

6	F	+	-	-	-	-	-	-	-	-	-	-	-	-	-	+	-	+

7	M	+	-	+	+	-	-	-	+	-	-	+	-	-	-	+	+	-

8	F	+	-	-	-	-	-	-	-	-	-	-	-	-	+	+	-	-

9	M	+	-	-	-	-	-	-	-	-	-	-	-	-	+	-	-	-

ID identification number of MCC patients; RCA, rolling circle amplification; LT1, LT3, M1/2, LT5, LT2 viral large T genes; VP1, VP1.3 viral caspid genes; P1, P3, P6, P9, P12 gene-specific primers for MCPyV detection by Feng et al. [5].

**Figure 2:**
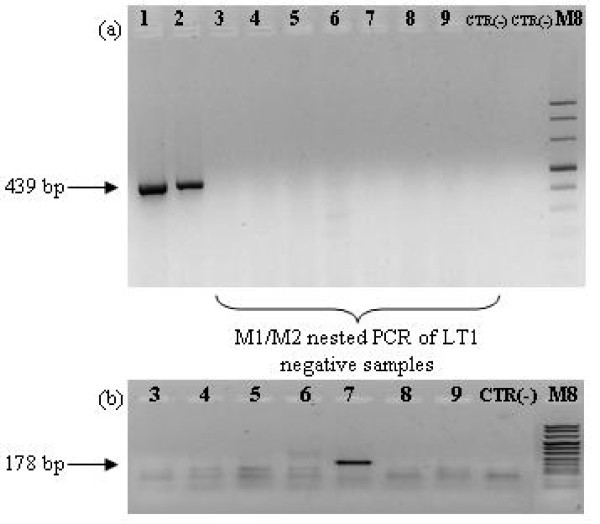
**RT-PCR of total RNA from MCC samples**. RT-PCR (a) was performed with gene-specific primers for LT1; nested PCR (b) was carried out with gene-specific primers (M1/2) within the LT1 amplicon. The numbers indicate the patients as in Table 1. M8, molecular weight marker (Roche, Milan, Italy); CTR (-), sterile water as negative control.

Amplifiable RNA was obtained for seven FFPE section specimens of the nine MCCs. RT-PCR with LT1 and M1/2 as nested primer sets revealed the presence of MCPyV RNA in 3 samples (Table 1, Figure 2). The sequences of the amplified products in all samples were exactly matching those of MCPyV type 339 [GenBank: EU375804.1]. In two out of nine MCPyV-positive MCCs, episomal viral DNA was detected by RCA (Table 1).

Taken together our data demonstrate that MCPyV is associated with MCC also in Italian patients, confirming the results of other studies [5,7,9,11,12] in different countries and stressing the possible role of MCPyV as an etiologic agent in the carcinogenesis of MCC. In addition, the presence of viral mRNAs in about 40% of tumours (3 out of 7 samples with amplifiable RNA) further supports the hypothesis that MCPyV plays a role in the molecular pathogenesis of MCC [5-12]. Data from RCA analyses lead to speculate that MCPyV is rarely detected as episome in these tumours suggesting, although not proving, that the virus could be integrated and this integration may precede the clonal expansion of tumour cells [5]. Finally confirmation of MCPyV as a contributing factor to the pathogenesis of MCC might provide novel options for future therapeutic strategies including immunotherapy.

## Competing interests

The authors declare that they have no competing interests.

## Authors' contributions

FP carried out the molecular genetic studies, participated in the sequence alignment and drafted the manuscript. AA contributed to the analysis and interpretation of data. PD contributed to the analysis, interpretation and acquisition of data. SB contributed to the analysis and interpretation of data. EM contributed to the analysis, interpretation and acquisition of data. AV conceived of the study, and participated in its design and coordination and helped to draft the manuscript. All authors read and approved the final manuscript.
